# Phenotypic Distinctions Between *EYS*- and *USH2A*-Associated Retinitis Pigmentosa in an Asian Population

**DOI:** 10.1167/tvst.14.2.16

**Published:** 2025-02-11

**Authors:** Ellis Y. H. Yeo, Taro Kominami, Tien-En Tan, Lathiksha Babu, Kevin G. S. Ong, Weilun Tan, Yasmin M. Bylstra, Kanika Jain, Rachael W. C. Tang, Saadia Z. Farooqui, Sylvia P. R. Kam, Choi-Mun Chan, Ranjana S. Mathur, Saumya S. Jamuar, Weng Khong Lim, Koji Nishiguchi, Beau J. Fenner

**Affiliations:** 1 Singapore National Eye Centre, Singapore; 2 Department of Ophthalmology, Nagoya University Hospital, Japan; 3 Singapore Eye Research Institute, Singapore; 4 Ophthalmology and Visual Sciences Academic Clinical Program, Duke-NUS Graduate Medical School, Singapore; 5 Institute for Precision Medicine, Duke-NUS Graduate Medical School, Singapore; 6 Genome Institute of Singapore, Singapore; 7 Genetics Service, Department of Paediatric Medicine, KK Women's and Children's Hospital, Singapore

**Keywords:** retinitis pigmentosa, inherited retinal disease, *EYS*, *USH2A*, gene therapy, genotype, east asia, usher syndrome

## Abstract

**Purpose:**

This study compares clinical characteristics of retinitis pigmentosa (RP) associated with mutations in the *EYS* and *USH2A* genes in a Southeast Asian cohort.

**Methods:**

Prospective single-center study of families with *EYS*- or *USH2A*-associated RP seen at the Singapore National Eye Centre. Comprehensive ophthalmic evaluations, multimodal imaging, genetic testing, and longitudinal follow-up identified clinically useful differentiating features between the two genotypes.

**Results:**

A total of 300 families with RP were enrolled, with *EYS*- and *USH2A*-associated RP, accounting for 24.7% of all probands and 50.7% of solved or likely solved cases. *USH2A* cases were predominantly nonsyndromic RP (75%). *EYS*-associated RP was more severe in functional and structural outcomes, and patients were more myopic than *USH2A* (SE −3.31 vs. −0.69; *P* < 0.0001). *EYS* RP displayed peripapillary nasal sparing on autofluorescence imaging more frequently than *USH2A* (57.6% vs. 26.7%; *P* = 0.006), whereas *USH2A* cases more often had a parafoveal ring (73.3% vs. 30.3%; *P* = 0.0002). Multiple logistic regression identified diagnostic features with 83.2% accuracy in distinguishing between *EYS* and *USH2A*, validated in a second unrelated clinical cohort.

**Conclusions:**

*EYS*- and *USH2A*-associated RP have overlapping clinical presentations but can often be distinguished based on a constellation of phenotypic features including disease onset and severity, refractive error, and fundus autofluorescence. These diagnostic features may support a more effective diagnostic strategy for these common forms of RP.

**Translational Relevance:**

Distinct clinical features differentiating *EYS*- and *USH2A*-associated RP provide valuable diagnostic tools that may inform personalized management and facilitate targeted interventions in clinical practice.

## Introduction

Pathogenic variants in the *EYS* (eyes shut homolog) and *USH2A* (Usher syndrome type 2A) genes are responsible for a substantial proportion of retinitis pigmentosa (RP) worldwide and are the most prevalent genetic causes of RP in East Asia.^1^^–^^8^ The coding sequences of both genes are exceptionally large—*EYS* is 9.4 kb and *USH2A* is 15.6 kb—and both are essential for structural integrity and function of photoreceptors,^9^^,^^10^ whereas *USH2A* is also important for inner ear cell function and is associated with both syndromic RP (Usher syndrome) and nonsyndromic autosomal recessive RP.^11^ The large size of both genes explains at least in part the high prevalence of RP secondary to variants in these genes,^12^ but presumed founder mutations such as *EYS* c.4957dupA (p.S1653fs) in Japanese^13^ and *EYS* c.6416G > A (p.C2139Y)^4^ and *USH2A* c.2802 T > G (p.C934W)^14^ in ethnic Chinese also influences the prevalence of RP caused by *EYS* and *USH2A* variants.

Retinal phenotypes of *EYS* and *USH2A*-associated RP have been described previously.^2^^,^^5^^,^^15^^–^^18^ Pathogenic variants in both genes cause generalized rod-cone degeneration, although atypical forms have been observed with sectoral and irregular distributions.^4^^,^^17^ Symptom onset varies between studies and in the case of *USH2A*, between syndromic and nonsyndromic disease, but has been reported to occur between the second to fourth decades of life,^14^^,^^19^^–^^21^ with widely variable median delays in presentation, from three to 24 years depending on the study.^19^^,^^20^^,^^22^^–^^24^ Cohort studies have demonstrated wide regional variability in presenting vision for both forms of RP, with median presenting best-corrected visual acuity (BCVA) ranging from logMAR 0.1 to 0.6.^14^^,^^20^^,^^22^^,^^23^ *USH2A*-associated RP has most commonly been reported as a typical generalized RP pattern of retinal involvement, whereas *EYS*-associated RP is associated with atypical retinal distributions that may be genotype-specific.^4^^,^^17^^,^^18^^,^^25^ There is also some evidence that patients with *EYS* is associated with myopia,^22^^,^^23^ although this is not uncommon among patients with RP.^26^ In general, however, there are no established phenotypic features that can reliably discriminate between these two prevalent forms of RP beyond the presence of congenital hearing loss in *USH2A*-associated Usher syndrome. This limitation is important in East Asia, where both *EYS* and *USH2A* are the most prevalent causes of RP, and standard diagnostic approaches such as exome sequencing may disclose a single genetic variant in either gene or multiple variants in both genes, confounding attempts to solve the genotype of an affected individual.

This prospective study aims to compare the clinical characteristics, disease progression, and genetic profiles of patients with *EYS*- and *USH2A*-associated RP. Conducted at tertiary eye centers in Singapore and Japan, this study enrolled patients diagnosed with RP and confirmed mutations in either the *EYS* or *USH2A* genes. Through comprehensive ophthalmic evaluations, genetic testing, and longitudinal follow-up, this research seeks to elucidate the differential impacts of these genetic mutations on retinal degeneration. The outcomes of this study aimed to provide insights into the natural history of *EYS*- and *USH2A*-associated RP, potentially informing personalized management strategies and future therapeutic developments.

## Methods

### Study Subjects

This was a prospective study of nonsyndromic RP patients with *EYS*- or *USH2A*-associated RP that were sequentially enrolled at the Singapore National Eye Centre, Singapore, during the period from March 2021 to December 2022, and at the Department of Ophthalmology at the Nagoya University Hospital. Patients with a clinical diagnosis of retinitis pigmentosa (RP), or rod-cone dystrophy, were included for further analysis. Diagnoses were made by fellowship-trained retinal specialists based on clinical history, physical examination, ophthalmic imaging, psychophysical testing, and genetic testing. The study was approved by the SingHealth and Nagoya University Hospital Institutional Review Boards and carried out in accordance with the ethical standards of the 1964 Declaration of Helsinki and its later amendments. Informed consent was obtained from all participants.

### Clinical Evaluation

All subjects underwent clinical evaluation, including a targeted clinical history, assessment of BCVA by logMAR testing, intraocular pressure measurement, and fundus examination by slit lamp biomicroscopy. Age at symptom onset (“onset”) was defined as the age at which a patient reported the onset of symptoms that were in the opinion of the managing clinician thought to be related to underlying retinitis pigmentosa (Table), whereas age at presentation (“presentation”) was the age at which a patient was initially seen in clinic. Color fundus photography (Triton DRI; Topcon Healthcare, Tokyo, Japan), ultrawide fundus autofluorescence imaging (Optos, Marlborough, MA, USA), macular optical coherence tomography (OCT; Spectralis; Heidelberg Engineering, Heidelberg, Germany), Ishihara color vision testing, and Goldmann kinetic perimetry (Haag-Streit, Koeniz, Switzerland) was performed on all subjects. The extent of visual field was measured as the extent of the V4e isopter (field diameter in degrees) at the horizontal midline as previously described.^4^ Foveal-centered horizontal line scans from macular OCT (30°) raster images were used for measurement of the horizontal diameter of ellipsoid bands, using the built-in Spectralis Eye Explorer software caliper tool (Heidelberg Engineering), as described in detail elsewhere.^27^ A value of zero was assigned if no discrete ellipsoid band was discernable. Horizontal and vertical visual field extent was measured as the number of degrees spanned by the V4e stimulus at the horizontal and vertical midlines on the Goldmann kinetic perimetry grid. Electroretinography was performed as an adjunctive test according to international society for clinical electrophysiology of vision (ISCEV) methodology were retained responses were anticipated based on clinical examination.^28^ Where indicated, the spherical equivalent (SE) was calculated in patients for whom phakic refraction data were available and for whom no history of refractive surgery was present at the time of refraction. Selected phenotypic features were scored in a second cohort from Nagoya, Japan, using clinical and retinal imaging data acquired the same approaches to that used in the primary study cohort. All patients completed psychophysical and structural testing, and a subset (n = 20 of 35 for *EYS*, n = 18 of 36 for *USH2A*) also completed electrophysiological testing.

**Table.** Baseline Characteristics of the *EYS*- and *USH2A*-Associated Nonsyndromic RP Cohorts

	*EYS* (*n* = 35)	*USH2A* (*n* = 36)	Significance of Difference (*P*)
Female gender	13 (37.1%)	25 (69.4%)	0.007^*^
Median age at symptom onset (IQR)	25.0 (16.0–45.0)	35.0 (15.25–49.5)	0.947
Median age at presentation (IQR)	38.5 (28.7–51.5)	39.5 (31.25–54.5)	0.864
Initial symptom (%)			
Nyctalopia	20 (57.1%)	24 (66.7%)	0.408
Reduced VA	7 (20%)	5 (13.9%)	0.496
Field loss	2 (5.7%)	3 (8.3%)	0.670
Incidental	4 (11.4%)	3 (8.3%)	0.663
Hemeralopia	2 (5.75)	0	0.149
Dyschromatopsia	0	1 (2.8%)	0.322
Chinese ethnicity	34 (97.1%)	35 (97.2%)	0.980
Median logMAR BCVA at presentation (IQR)	0.2 (0.1–0.4)	0.18 (0.1–0.3)	0.515
Median horizontal field at presentation (IQR)	65.0 (36.0–123.5)	83.5 (35.8–128)	0.874
Median horizontal EZ width (µm) at presentation (IQR)	2482 (703–4938)	2421 (1205–3973)	0.993
Median follow-up (IQR)	11.4 (2.1–15.5)	7.3 (4.3–14.4)	0.917

Comparisons were made using the Mann-Whitney test for continuous variables and χ^2^ test of proportions for categorical data.

* Represents values with *P* < 0.05

### Genetic Analyses

DNA was obtained from peripheral blood leukocytes according to standard procedures. Whole exome sequencing (WES) was performed by Macrogen (Geumcheon-gu, Seoul, South Korea) and Novogene (Singapore), and bioinformatics analysis of sequence data was performed at the SingHealth Duke-NUS Institute of Precision Medicine (PRISM), Singapore. When necessary, direct sequencing was performed with the BigDye Terminator Cycle Sequencing kit (Applied Biosystems, Foster City, CA, USA) with 10 ng of template DNA in each reaction and using a PCR program of 25 cycles of denaturation at 97°C for 30 seconds, annealing at 50°C for 15 seconds, and extension at 60°C for four minutes. Samples were analyzed in a 3130 Genetic Analyzer (Applied Biosystems). Genetic variant calls were made by comparison with SG10K,^29^ a Singapore population-based genome reference database, with additional reference to the gnomAD genomic database^30^ and ClinVar^31^^,^^32^ for variants absent from SG10K. Variant phase was established by direct sequencing of the relevant variants in one or more unaffected first-degree relatives. The gnomAD genomic database^20^ was queried to identify variants that were classified as pathogenic or likely pathogenic according to annotations from the ClinVar variant database. Determination of variant pathogenicity was based on criteria from the American College of Medical Genetics and Genomics. A subset of patients underwent panel-based clinical exome sequencing, performed by Molecular Vision Laboratories (Hillsboro, OR, USA), and these patients were also enrolled into the study and their clinical and genetic data analyzed with the research testing cohort. Genetic variant data are provided in Supplementary Tables S1, S2, and S3.

### Statistical Analyses

Statistical analyses were performed using Prism 10.2 (GraphPad Software, Boston, MA, USA). Comparisons between clinical subgroups were performed using Mann-Whitney (pairwise) and Kruskal-Wallis tests (>2 group analyses). LogMAR visual acuity values of 1.9, 2.3, 2.7, and 3.0 were used to represent counting fingers, hand motions, light perception, and no light perception, respectively, for the purposes of statistical comparisons. Kaplan Meier survival analyses and Mantel Cox log-rank tests were performed using the specified endpoints with ages at which patients reach each endpoint being imputed for analysis. Regression analyses used to correlate spherical equivalent with age at onset were performed using *t* tests for slope, with significance set at *P* < 0.05. Simple linear regression was used to compare changes in BCVA, goldmann visual field (GVF), and OCT outcomes between groups over time (age and years since symptom onset). Regression lines were compared using analysis of covariance (ANCOVA). Multiple logistic regression was performed following conversion of clinical features into categorical variables, whereby continuous variables, including age at symptom onset, spherical equivalent in diopters, and presenting logMAR BCVA, were binned according to the median value for each variable after pooling data from *EYS* and *USH2A* cases.

## Results

**Figure 1.**
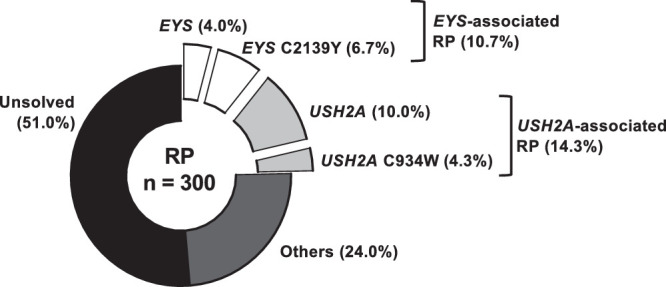
Composition of RP genotypes in the Singapore RP study cohort. A total of 300 unrelated RP probands were included. Cases with biallelic *EYS* or *USH2A* RP, biallelic *EYS* or *USH2A* RP wherein one or both alleles included the *EYS* C2139Y or *USH2A* C934W variant or were only a single *EYS* or *USH2A* variant was identified, are shown in exploded slices. The remaining RP cases without *EYS* or *USH2A*, both solved/others and unsolved, are also shown.

**Figure 2.**
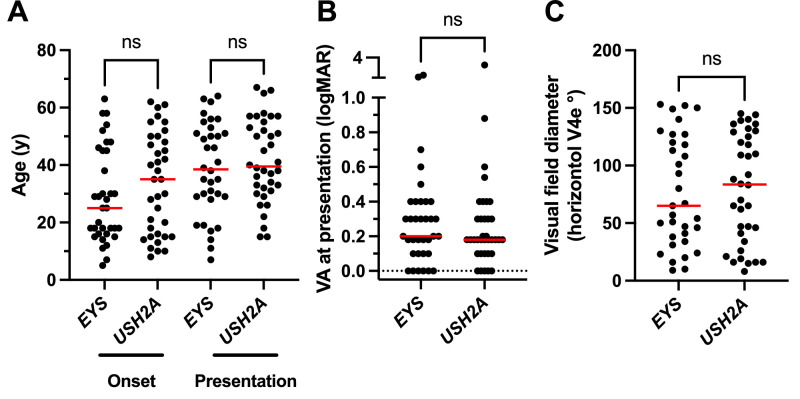
Baseline clinical features of RP cases with *EYS* or *USH2A*. (**A**) Age at symptom onset and presentation; (**B**) BCVA; and (**C**) horizontal visual field diameter (°) on the V4e isopter on Goldmann kinetic perimetry are sown as scatter plots. Median values are shown as *horizontal bars*. *EYS* and *USH2A* groups were compared with Mann Whitney tests. Ns, not significant.

We included 300 unrelated individuals with RP in the initial cohort (Fig. 1), for whom 48.7% had a solved or likely solved disease-causing genotype. Collectively, *EYS* and *USH2A* accounted for 24.7% of all probands with RP and 50.7% of probands with solved or likely solved genotypes. Baseline characteristics of patients with nonsyndromic *EYS-* and *USH2A*-associated RP are described in the Table. Individuals with biallelic *EYS*- and *USH2A*-associated RP comprised 10.7% (n = 32 families; 35 affected individuals) and 14.3% (n = 43 families; 48 affected individuals) of families enrolled and genotyped during the study period, respectively (Fig. 1). Among *EYS* probands, 62.5% (n = 20) contained the common variant *EYS* c.6416G > A (p.C2139Y), whereas the common *USH2A* variant c.2802T > G (p.C934W) was present in 14.3 (n = 13) of *USH2A* probands. Usher syndrome was present in 12 (25%) individuals in the *USH2A* cohort, and these individuals were excluded from subsequent analysis. There were significantly more females in the *USH2A* cohort compared to the *EYS* cohort (62.5% vs. 37.1%; *P* = 0.007). Symptom onset occurred far earlier in patients with *EYS* compared to *USH2A* (median onset 25.0 and 35.0; *P* = 0.947; Fig. 2A), although limited sample sizes meant that this did not reach statistical significance.

**Figure 3.**
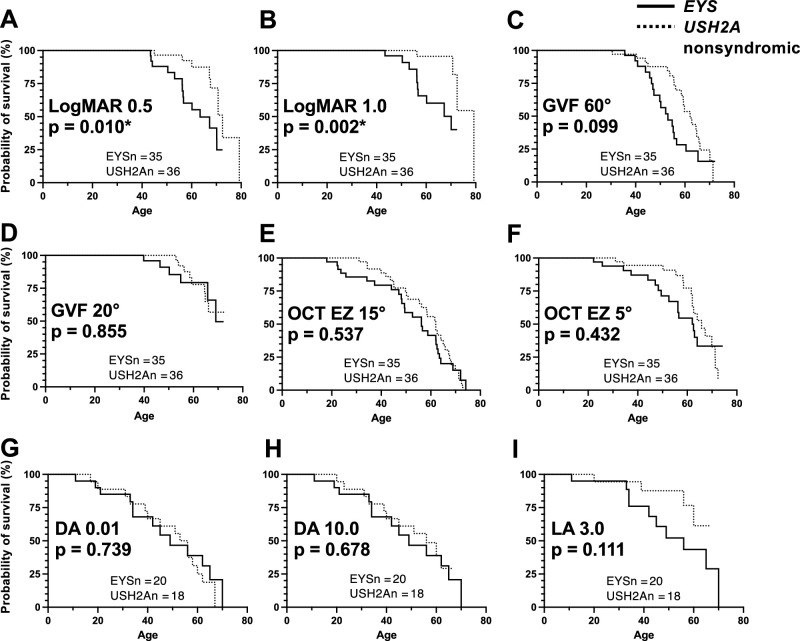
Survival curves for *EYS*- and *USH2A*-associated RP clinical features. Visual acuity cutoffs of logMAR 0.5 (**A**) and 1.0 (**B**), visual field cutoffs of 60° (**C**) and 20° (**D**), ellipsoid band width of 15° (**E**) and 5° (**F**), and ffERG extinction for DA 0.01 (**G**), DA 10.0 (**H**), and LA 3.0 (**I**) are shown for both groups. Survival curves were compared using log-rank (Mantel Cox) tests for significance, with *P* values shown as insets.

Both groups initially presented at a similar age—median age at presentation for *EYS* was 38.5 and *USH2A* was 39.5 (*P* = 0.864; Fig. 2A). The median delay between onset of symptoms and presentation to the clinic was 9.49 years (interquartile range [IQR] 0.35–24.8 years) for *EYS* and 11.5 years (IQR 3.1–23.7 years) for *USH2A*, which was comparable between the groups (Mann-Whitney, *P* = 0.445). Presenting visual acuity and the horizontal diameter of the visual field (measured in degrees on GVF, V4e isopter) was also better in patients with *USH2A* compared to *EYS*, but this was not statistically significant (Figs. 2B, 2C). We further examined the two cohorts to compare the natural history of disease progression between the two cohorts and identify clinically useful phenotypic features that could be used to distinguish between the two genotypes. Survival curve analysis for BCVA of logMAR 0.5 (Snellen 20/60) and logMAR 1.0 (Snellen 20/200) revealed significant separation of the *EYS* and *USH2A* groups, with *EYS* median survival lower than *USH2A* in both cases (Figs. 3A, 3B). Median survival for logMAR 0.5 was 63.5 years for *EYS* and 72.5 years for *USH2A* (*p* = 0.010), and for logMAR 1.0 median survival was 70.1 and 79.3 for *EYS* and nonsyndromic *USH2A*, respectively (*P* = 0.002). Other structural and functional outcomes, including loss of visual fields, macular ellipsoid band loss, and ffERG extinction, were similar between *EYS* and *USH2A*, although there was a clear trend toward *EYS* being more rapidly progressive than *USH2A* (Fig. 3). Only a subset of cases (n = 20 for *EYS* and n = 18 for *USH2A*) underwent ffERG testing based on discretion of the managing clinician, so a selection bias for this aspect of the comparison cannot be excluded. Despite this, it appeared quite clear from BCVA, visual fields, OCT, and ERG metrics that *EYS* was the more severe and rapidly progressive disease.

**Figure 4.**
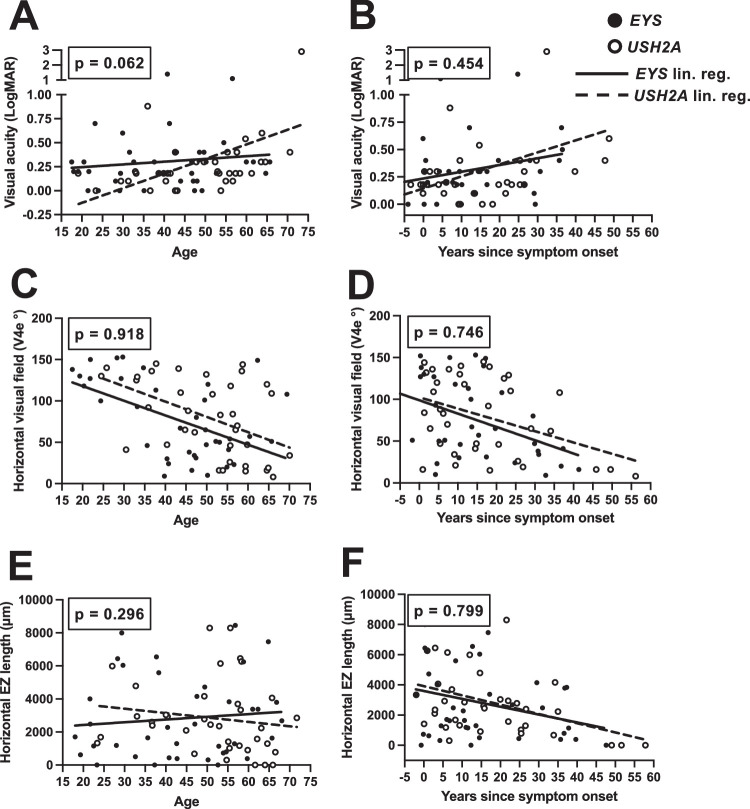
Comparisons in outcomes for *EYS*- and *USH2A*-associated RP, based in patient age and time elapsed since symptom onset. Presenting BCVA (**A** and **B**), horizontal visual field diameter (**C** and **D**), and horizontal EZ band length (**E** and **F**) are plotted against patient age or years elapsed since symptom onset. Linear regression (lin. reg.) was used to compare the two genotypes, with ANCOVA analysis comparing the regression lines. Significance of ANCOVA is shown as *P* values inset.

Regression analysis of visual fields, visual acuity, and EZ band width as a function of patient age and years since symptom onset showed similar rates of functional and structural decline in *EYS* and *USH2A*, with years since symptom onset demonstrating better correlation over time than patient age (Fig. 4). Unlike the survival curve analyses in Figure 3, where *EYS* was clearly more severe than *USH2A*, the regression approach did not clearly separate two diseases.

We next examined additional baseline features that differed between the *EYS* and *USH2A* groups. Patients with *EYS* were significantly more myopic than patients with *USH2A* (SE −3.38 vs. −0.68; *P* < 0.0001; Supplementary Fig. S1). We hypothesized that the earlier onset of disease among *EYS* cases, with attendant loss of photoreceptors and potential disruption of normal emmetropization, may have contributed to higher myopia in *EYS* cases. There was a modest but significant correlation between age at symptom onset and myopia for *USH2A* patients, with earlier onset of symptoms being associated with higher myopia (*r*^2^ = 0.1; *P* = 0.037; Supplementary Fig. S1). However, there was no correlation between symptom onset and myopia for *EYS* (*r*^2^ = 0.009; *P* = 0.607; Supplementary Fig. S1). We also excluded a possible gender effect on myopia by separately comparing males and females with *EYS* and *USH2A* (Supplementary Fig. S2), but the significant finding of higher myopia in patient with *EYS*-associated RP remained.

**Figure 5.**
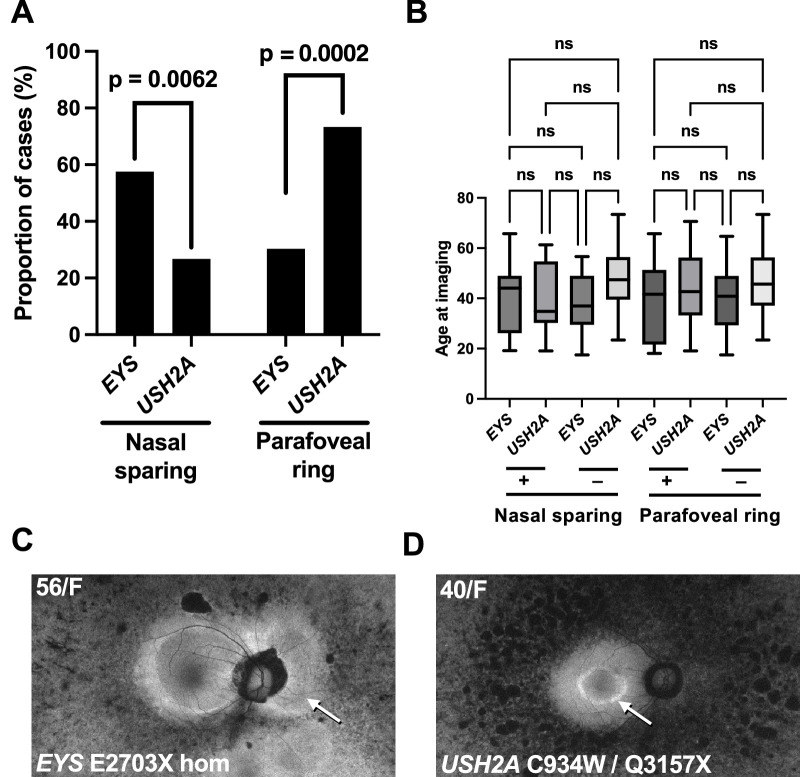
Autofluorescence imaging distinguishes between *EYS* and *USH2A* RP. (**A**) Peripapillary nasal sparing was more prevalent among individuals with *EYS* (*P* = 0.0019) whereas a parafoveal ring was more prevalent among individuals with *USH2A* (*P* = 0.0002). (**B**) Illustrative cases for both features are shown, along with the *EYS* and *USH2A* genotypes identified for each case.

Fundus autofluorescence (AF) patterns are useful for the diagnosis and characterization of inherited retinal diseases, and *EYS*-associated RP is known to have atypical patterns of AF.^4^^,^^17^^,^^18^ We thus reviewed the ultra-widefield AF patterns of the *EYS* and *USH2A* cohorts to identify distinguishing features. Two features, namely sparing of the peripapillary retina and the presence of a parafoveal hyperautofluorescent ring (the so-called *Robson-Holder ring*) were identified as potentially discriminating features between the two genotypes. Peripapillary nasal sparing was observed in 57.6% of *EYS* cases, compared to 26.7% of *USH2A* cases (*P* = 0.006; Fig. 5). Conversely, a parafoveal ring was observed in 30.3% of *EYS* cases but occurred in 73.3% of *USH2A* cases (*P* = 0.0002; Fig. 5). Given the progressive nature of RP and changes in AF patterns over time in a particular eye, we compared the ages of patients with nasal sparing and parafoveal ring to patients without these signs (Fig. 5). No significant difference in age was observed between individuals with and without the AF features, in both the *EYS* and *USH2A* cohorts. Logistic regression analysis demonstrated that an *USH2A* genotype was associated with a parafoveal ring (*z* value 3.443; *P* = 0.001) independent of patient age and ellipsoid band width (Supplementary Table S4), whereas an *EYS* genotype (*z* value 2.802; *P* = 0.005) and the ellipsoid band width (*z* value 3.352; *P* = 0.001) were independently associated with the presence nasal sparing (Supplementary Table S5).

**Figure 6.**
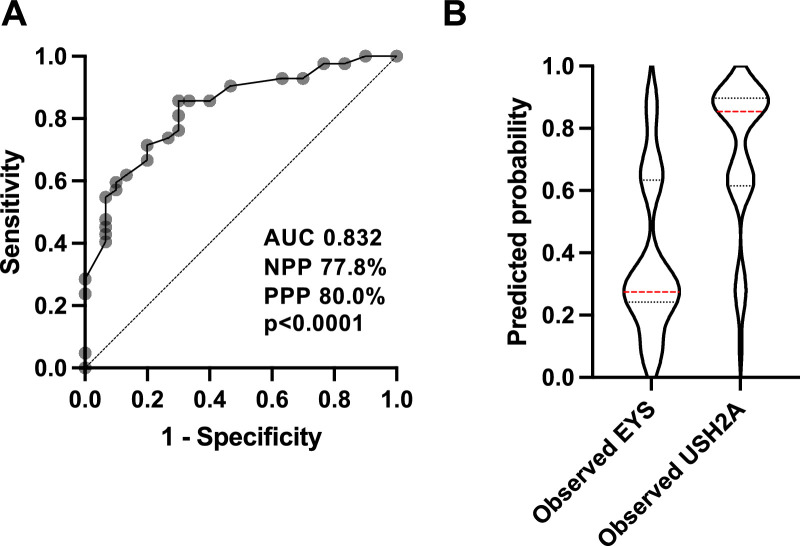
Multiple logistic regression analysis incorporating age at symptom onset, presenting BCVA, spherical equivalent, peripapillary nasal sparing, and perifoveal ring or fundus AF, were used to distinguish between *EYS* and *USH2A* (**A**). Negative predictive power (NPP) and positive predictive power (PPP) are shown. (**B**) The predicted probabilities for *EYS* and *USH2A* cohorts are shown as violin plots, where median and quartiles are shown as horizontal bars.

We pooled our findings for each of the potentially discriminating features for *EYS* and *USH2A* to determine whether a combination of phenotypic features would be clinically useful for distinguishing between the two genotypes. Cases with *USH2A*-associated Usher syndrome were excluded from this analysis. Multiple logistic regression identified five useful diagnostic features, namely age at symptom onset (<28 years), spherical equivalent (≤−2D), presenting BCVA (>0.2 logMAR), absent parafoveal ring on AF, and presence of nasal peripapillary sparing on AF. When combined these features yielded a diagnostic accuracy of 83.2% for distinguishing between *EYS*- and *USH2A*-associated RP, with a negative predictive power of 77.8% and positive predictive power of 80.0% (Fig. 6). The weightings of each clinical feature are described in Supplementary Table S6.

Finally, we tested the clinical utility of the model with a second cohort of patients previously enrolled at the University of Nagoya in Japan. For this test we scored each of the distinguishing features for 10 patients with *EYS* and nine patients with *USH2A*. In total, eight of 10 *EYS* and eight of nine *USH2A* cases were corrected predicted. This amounted to an accuracy of 84.2%, sensitivity (recall) of 80%, specificity of 88.9%, precision of 88.9%, and an F1 score (harmonic mean of precision and recall) of 84.2% for prediction of an *EYS* or *USH2A* genotype.

## Discussion

RP associated with variants in *EYS* and *USH2A* is by far the most common cause of RP in East Asia, and this was particularly evident in the current study, where *EYS* and *USH2A* were collectively responsible for more than half of RP cases with an identifiable disease-causing genotype. Distinguishing between these two genetic forms of RP is clinically useful both in Singapore and other East Asian nations, given their high prevalence and the implications for diagnosis, genetic counseling, and potential therapies. Our findings demonstrate clear differences in the phenotypic characteristics of these two forms of RP, contributing knowledge to the understanding of their natural history and clinical manifestations. We found that the age of symptom onset was generally earlier for *EYS*-associated RP, with our findings broadly aligning with previous studies reporting similar age ranges for symptom onset in East Asian populations. Japanese patients with *EYS* mutations presented with symptoms in their mid-20s,^3^ compared to age 27 for Chinese patients with *USH2A* mutations.^2^ Both findings are consistent with our cohort's median onset age of 25 years for *EYS* and 28 years for *USH2A*, respectively. Although both groups exhibited comparable baseline visual acuity and horizontal visual field extents, survival curve analysis demonstrated that *EYS* was the more severe of the two genotypes across a range of functional and structural outcomes. However, our study also identified specific phenotypic markers that distinguish the two groups. Patients with *EYS*-associated RP were more myopic, and distinct fundus AF patterns differed between the groups, findings that have not been consistently emphasized in prior studies.

AF patterns are potentially useful for diagnosing and characterizing inherited retinal diseases.^33^^–^^35^ Our observation that *EYS*-associated RP commonly exhibits peripapillary nasal sparing corroborates earlier work^16^^,^^17^ documenting unusual AF features among these individuals. Recent in vitro studies of *EYS* suggest that the EYS protein may play a role in photoprotection.^9^ This potentially explains the atypical patterns of retinal degeneration in a subset of patients with *EYS* RP, wherein loss of EYS function results in retinal phototoxicity and degeneration.

The high prevalence of *EYS* c.6416G > A (p.C2139Y) and *USH2A* c.2802T > G (p.C934W) variants in our cohort is consistent with reports of presumed founder mutations in ethnic Chinese populations. Lin and colleagues^14^ recently reported on the high prevalence of *USH2A* c.2802T > G in Taiwanese patients with RP, while we previously demonstrated prevalence of *EYS* c.6416G > A among ethnic Chinese in Singapore.^4^ The precise geographic origins of these two genetic variants remain indeterminate, although both appear to be common among ethnic Chinese, with the *EYS* c.6416G > A variant in particular being prevalent among individuals of Southern Chinese descent. Interestingly, a recent analysis by the Human Genome Diversity Project identified *EYS* c.6416G > A exclusively in the She Chinese ancestral group that arose from Southern China.^30^ This contrasts with *USH2A* c.2802T > G, which was present among ancestral groups from Central and Northern China, Mongolia, the Sakha region of Russia, and to a lesser extent Japanese.^30^ This may explain the higher prevalence of *EYS* c.6416G > A and relatively lower prevalence of *USH2A* c.2802T > G among Singaporean Chinese, who are predominantly of Southern Chinese descent. The potentially large number of individuals affected by RP involving these variants makes both extremely attractive as gene therapy targets.^4^ Indeed, Pongpaksupasin and colleagues^36^ recently developed a retinal stem cell line with *EYS* c.6416G > A, which may prove a useful model for testing targeted therapies.

Our findings have several important implications for clinical practice and research. The identification of phenotypic markers such as myopia and specific AF patterns provides valuable diagnostic tools for distinguishing between *EYS*- and *USH2A*-associated RP. The association between *EYS* and myopia, independent of age at symptom onset, is particularly notable. Myopia in RP ostensibly occurs during childhood, with severe or early onset RP, such as *RPGR*-associated RP, being known to associate with myopia.^26^ However, we were unable to demonstrate a clear association between symptom onset and myopia with *EYS*-associated RP. One possible explanation for this is that the *EYS* gene may play a role in the structural integrity of the photoreceptors, influencing refractive development. Our findings suggest that *EYS*-associated RP is clearly associated with myopia, while *USH2A* cases did not appear to be significantly myopic. This might be due to differences in the underlying pathophysiology of photoreceptor degeneration between the two genes.

The diagnostic utility of the clinical phenotypes we identified is another important consideration. The five features identified here yielded good diagnostic accuracy and are clinically facile. Unlike black-box deep learning approaches, the phenotypic features described here are easily applied using widely available tools. These phenotypic features may be particularly useful in unsolved cases with incomplete genotypes where cases with strong suspicion based on clinical and imaging features may be prioritized for genome or long-read sequencing to solve incomplete *EYS* or *USH2A* genotypes.

We acknowledge several important limitations of the current work. The cohort size, while representative, may limit the generalizability of the findings to other regional populations. We did demonstrate clinical utility of several phenotypic features for *EYS* and *USH2A* RP in a Japanese cohort distinct from the primary study cohort from Singapore, but it remains to be seen if these features will carry over to, for example, European and U.S.-based cohorts. We would also like to emphasize that our use of fundus autofluorescence, while potentially useful as one of several phenotypic features to might distinguish between *EYS* and *USH2A*, is limited by the transient nature of autofluorescent fundus features observed in RP. Nasal sparing and the parafoveal ring seen on autofluorescence are expected to exist only during a specific period for any given patient,^37^ so their use may not be relevant in very early or very late-stage disease. Both signs are also by no means genotype-specific and can appear across a wide range of RP genotypes.^38^ Larger multi-center studies are needed to confirm our results and extend them to additional populations. Moreover, larger cohorts would enable better delineation of the clinical differences in *EYS*- and *USH2A*-associated RP. An obvious trend towards milder disease was observed in individuals with *USH2A*-associated RP, and it is likely that this difference would become significant with increased cohort sizes. It is also clear after reviewing the genotypes of our cohort that our results likely exhibit regional or ethnic bias. The large proportion of recurrent variants in the *EYS* and *USH2A* cohorts would exert a significant impact on any comparison made between the groups and potentially limit the generalizability to other geographic regions or ethnic groups with differing genetic structures.

Exploring innovative gene therapy approaches that can overcome the challenges posed by the large size of *EYS* and *USH2A* genes will be crucial. Current gene therapy strategies are limited by the packaging capacity of adeno-associated viral vectors, commonly used for retinal gene therapy. Dual-vector strategies, which split the gene into two separate adeno-associated viral vectors, or the use of larger viral vectors such as lentivirus, could potentially address this issue. Additionally, gene editing technologies such as CRISPR/Cas9 offer promising alternatives for correcting mutations in large genes.^39^ This study reports on the first comparative study of the two most common causes of RP in East Asia. The identification of distinctive phenotypic markers and progression patterns for *EYS*- and *USH2A*-associated RP offers practical diagnostic tools and highlights areas for future research and therapeutic development.

## Supplementary Material

Supplement 1

Supplement 2

Supplement 3

Supplement 4

Supplement 5

Supplement 6

Supplement 7

Supplement 8

## References

[1] Ng TK, Tang W, Cao Y, et al. Whole exome sequencing identifies novel USH2A mutations and confirms Usher syndrome 2 diagnosis in Chinese retinitis pigmentosa patients. *Sci Rep*. 2019; 9: 5628. 30948794 10.1038/s41598-019-42105-0 PMC6449333

[2] Gao FJ, Wang DD, Chen F, et al. Prevalence and genetic-phenotypic characteristics of patients with USH2A mutations in a large cohort of Chinese patients with inherited retinal disease. *Br J Ophthalmol*. 2021; 105: 87–92. 32188678 10.1136/bjophthalmol-2020-315878 PMC7788223

[3] Arai Y, Maeda A, Hirami Y, et al. Retinitis Pigmentosa with EYS Mutations Is the Most Prevalent Inherited Retinal Dystrophy in Japanese Populations. *J Ophthalmol*. 2015; 2015: 819760. 26161267 10.1155/2015/819760 PMC4487330

[4] Chan CM, Tan TE, Jain K, et al. Retinitis pigmentosa associated with the EYS C2139Y variant: an important cause of blindness in East Asian populations. *Retina*. 2023. 10.1097/IAE.0000000000003874 37418643

[5] Zhu T, Chen DF, Wang L, et al. USH2A variants in Chinese patients with Usher syndrome type II and non-syndromic retinitis pigmentosa. *Br J Ophthalmol*. 2021; 105: 694–703. 32675063 10.1136/bjophthalmol-2019-315786

[6] Gao FJ, Wang DD, Hu FY, et al. Genotypic spectrum and phenotype correlations of EYS-associated disease in a Chinese cohort. *Eye (Lond)*. 2022; 36: 2122–2129. 34689181 10.1038/s41433-021-01794-6 PMC9581949

[7] Xiao X, Cao Y, Chen S, et al. Whole exome sequencing reveals novel EYS mutations in Chinese patients with autosomal recessive retinitis pigmentosa. *Mol Vis*. 2019; 25: 35–46. 30804660 PMC6363637

[8] Numa S, Oishi A, Higasa K, et al. EYS is a major gene involved in retinitis pigmentosa in Japan: genetic landscapes revealed by stepwise genetic screening. *Sci Rep*. 2020; 10: 20770. 33247286 10.1038/s41598-020-77558-1 PMC7695703

[9] Otsuka Y, Imamura K, Oishi A, et al. Phototoxicity avoidance is a potential therapeutic approach for retinal dystrophy caused by EYS dysfunction. *JCI Insight*. 2024; 9. 10.1172/jci.insight.174179 PMC11141876 38646933

[10] Stemerdink M, García-Bohórquez B, Schellens R, Garcia-Garcia G, Van Wijk E, Millan JM. Genetics, pathogenesis and therapeutic developments for Usher syndrome type 2. *Hum Genet*. 2022; 141: 737–758. 34331125 10.1007/s00439-021-02324-w

[11] Toms M, Pagarkar W, Moosajee M. Usher syndrome: clinical features, molecular genetics and advancing therapeutics. *Ther Adv Ophthalmol*. 2020; 12: 2515841420952194. 32995707 10.1177/2515841420952194 PMC7502997

[12] Pontikos N, Arno G, Jurkute N, et al. Genetic Basis of Inherited Retinal Disease in a Molecularly Characterized Cohort of More Than 3000 Families from the United Kingdom. *Ophthalmology*. 2020; 127: 1384–1394. 32423767 10.1016/j.ophtha.2020.04.008 PMC7520514

[13] Iwanami M, Oshikawa M, Nishida T, Nakadomari S, Kato S. High prevalence of mutations in the EYS gene in Japanese patients with autosomal recessive retinitis pigmentosa. *Invest Ophthalmol Vis Sci*. 2012; 53: 1033–1040. 22302105 10.1167/iovs.11-9048

[14] Lin YW, Huang YS, Lin CY, et al. High prevalence of exon-13 variants in USH2A-related retinal dystrophies in Taiwanese population. *Orphanet J Rare Dis*. 2024; 19: 238. 38879497 10.1186/s13023-024-03238-2 PMC11179209

[15] Iwanami M, Oishi A, Ogino K, et al. Five major sequence variants and copy number variants in the EYS gene account for one-third of Japanese patients with autosomal recessive and simplex retinitis pigmentosa. *Mol Vis*. 2019; 25: 766–779. 31814702 PMC6857781

[16] Mucciolo DP, Sodi A, Passerini I, et al. Fundus phenotype in retinitis pigmentosa associated with EYS mutations. *Ophthalmic Genet*. 2018; 39: 589–602. 30153090 10.1080/13816810.2018.1509351

[17] Sengillo JD, Lee W, Nagasaki T, et al. A distinct phenotype of eyes shut homolog (EYS)-retinitis pigmentosa is associated with variants near the C-terminus. *Am J Ophthalmol*. 2018; 190: 99–112. 29550188 10.1016/j.ajo.2018.03.008 PMC8451245

[18] Soares RM, Carvalho AL, Simão S, et al. Eyes shut homolog-associated retinal degeneration: natural history, genetic landscape, and phenotypic spectrum. *Ophthalmol Retina*. 2023; 7: 628–638. 36764454 10.1016/j.oret.2023.02.001

[19] Suvannaboon R, Pawestri AR, Jinda W, Tuekprakhon A, Trinavarat A, Atchaneeyasakul LO. Genotypic and phenotypic profiles of EYS gene-related retinitis pigmentosa: a retrospective study. *Sci Rep*. 2022; 12: 21494. 36513702 10.1038/s41598-022-26017-0 PMC9748023

[20] Placidi G, Maltese PE, Savastano MC, et al. Retinitis pigmentosa associated with EYS gene mutations: disease severity staging and central retina atrophy. *Diagnostics (Basel)*. 2023; 13: 850. 36899994 10.3390/diagnostics13050850 PMC10000790

[21] Birch DG, Cheng P, Duncan JL, et al. The RUSH2A Study: best-corrected visual acuity, full-field electroretinography amplitudes, and full-field stimulus thresholds at baseline. *Transl Vis Sci Technol*. 2020; 9: 9. 10.1167/tvst.9.11.9 PMC7552938 33133772

[22] McGuigan DB, Heon E, Cideciyan AV, et al. EYS mutations causing autosomal recessive retinitis pigmentosa: changes of retinal structure and function with disease progression. *Genes (Basel)*. 2017; 8(7): 178. 28704921 10.3390/genes8070178 PMC5541311

[23] Bandah-Rozenfeld D, Littink KW, Ben-Yosef T, et al. Novel null mutations in the EYS gene are a frequent cause of autosomal recessive retinitis pigmentosa in the Israeli population. *Invest Ophthalmol Vis Sci*. 2010; 51: 4387–4394. 20375346 10.1167/iovs.09-4732

[24] Cundy O, Broadgate S, Halford S, et al. Genetic and clinical findings in an ethnically diverse retinitis pigmentosa cohort associated with pathogenic variants in EYS. *Eye (Lond)*. 2021; 35: 1440–1449. 32728228 10.1038/s41433-020-1105-8 PMC8182807

[25] Lo JE, Cheng CY, Yang CH, et al. Genotypes influence clinical progression in EYS-associated retinitis pigmentosa. *Transl Vis Sci Technol*. 2022; 11: 6. 10.1167/tvst.11.7.6 PMC9284463 35816039

[26] Hendriks M, Verhoeven VJM, Buitendijk GHS, et al. Development of refractive errors-what can we learn from inherited retinal dystrophies? *Am J Ophthalmol*. 2017; 182: 81–89. 28751151 10.1016/j.ajo.2017.07.008

[27] Ng LYB, Ang CZ, Tan TE, et al. When do patients with retinitis pigmentosa present to ophthalmologists? A multi-centre retrospective study. *Eye (Lond)*. 2024; 38: 3595–3600. 39322768 10.1038/s41433-024-03368-8 PMC11621706

[28] Robson AG, Frishman LJ, Grigg J, et al. ISCEV Standard for full-field clinical electroretinography (2022 update). *Doc Ophthalmol*. 2022; 144: 165–177. 35511377 10.1007/s10633-022-09872-0 PMC9192408

[29] Chan SH, Bylstra Y, Teo JX, et al. Analysis of clinically relevant variants from ancestrally diverse Asian genomes. *Nat Commun*. 2022; 13: 6694. 36335097 10.1038/s41467-022-34116-9 PMC9637116

[30] Karczewski KJ, Francioli LC, Tiao G, et al. The mutational constraint spectrum quantified from variation in 141,456 humans. *Nature*. 2020; 581: 434–443. 32461654 10.1038/s41586-020-2308-7 PMC7334197

[31] Landrum MJ, Lee JM, Benson M, et al. ClinVar: public archive of interpretations of clinically relevant variants. *Nucleic Acids Res*. 2016; 44: D862–868. 26582918 10.1093/nar/gkv1222 PMC4702865

[32] Landrum MJ, Lee JM, Riley GR, et al. ClinVar: public archive of relationships among sequence variation and human phenotype. *Nucleic Acids Res*. 2014; 42: D980–985. 24234437 10.1093/nar/gkt1113 PMC3965032

[33] Fujinami-Yokokawa Y, Ninomiya H, Liu X, et al. Prediction of causative genes in inherited retinal disorder from fundus photography and autofluorescence imaging using deep learning techniques. *Br J Ophthalmol*. 2021; 105: 1272–1279. 33879469 10.1136/bjophthalmol-2020-318544 PMC8380883

[34] Miere A, Le Meur T, Bitton K, et al. Deep learning-based classification of inherited retinal diseases using fundus autofluorescence. *J Clin Med*. 2020; 9: 3303. 33066661 10.3390/jcm9103303 PMC7602508

[35] Veturi YA, Woof W, Lazebnik T, et al. SynthEye: investigating the impact of synthetic data on artificial intelligence-assisted gene diagnosis of inherited retinal disease. *Ophthalmol Sci*. 2023; 3: 100258. 36685715 10.1016/j.xops.2022.100258 PMC9852957

[36] Pongpaksupasin P, Tong-Ngam P, Jearawiriyapaisarn N, et al. Generation of an EYS-associated retinitis pigmentosa patient-derived human pluripotent stem cell line (MUi038-A). *Stem Cell Res*. 2024; 78: 103448. 38810502 10.1016/j.scr.2024.103448

[37] Antropoli A, Arrigo A, Bianco L, Cavallari E, Bandello F, Battaglia Parodi M. Hyperautofluorescent ring pattern in retinitis pigmentosa: clinical implications and modifications over time. *Invest Ophthalmol Vis Sci*. 2023; 64: 8. 10.1167/iovs.64.12.8 PMC10484024 37669062

[38] Lee J, Asano S, Inoue T, et al. Investigating the usefulness of fundus autofluorescence in retinitis pigmentosa. *Ophthalmol Retina*. 2018; 2: 1062–1070. 31047495 10.1016/j.oret.2018.03.007

[39] Fenner BJ, Tan TE, Barathi AV, et al. Gene-based therapeutics for inherited retinal diseases. *Front Genet*. 2021; 12: 794805. 35069693 10.3389/fgene.2021.794805 PMC8782148

