# Supplementary material for: Phenotypic Distinctions Between EYS- and USH2A-Associated Retinitis Pigmentosa in an Asian Population
Source: Transl Vis Sci Technol. 2025 Feb 11;14(2):16. doi: 10.1167/tvst.14.2.16 (PMC11817848; doi:10.1167/tvst.14.2.16)

Supplementary Figure 1. Patients with *EYS*-associated RP are more myopic than individuals with *USH2A*-associated RP. (A) The spherical equivalent (SE) of individuals sampled from the general population and those with *EYS* and *USH2A* were compared via a Mann-Whitney test. Significance (****), p<0.0001. Linear regression analysis of *EYS* (B) and *USH2A* (C) cohorts showing association between age at symptom onset and the SE of patients. No significant association was identified for *EYS*, while *USH2A* had a significant positive association.

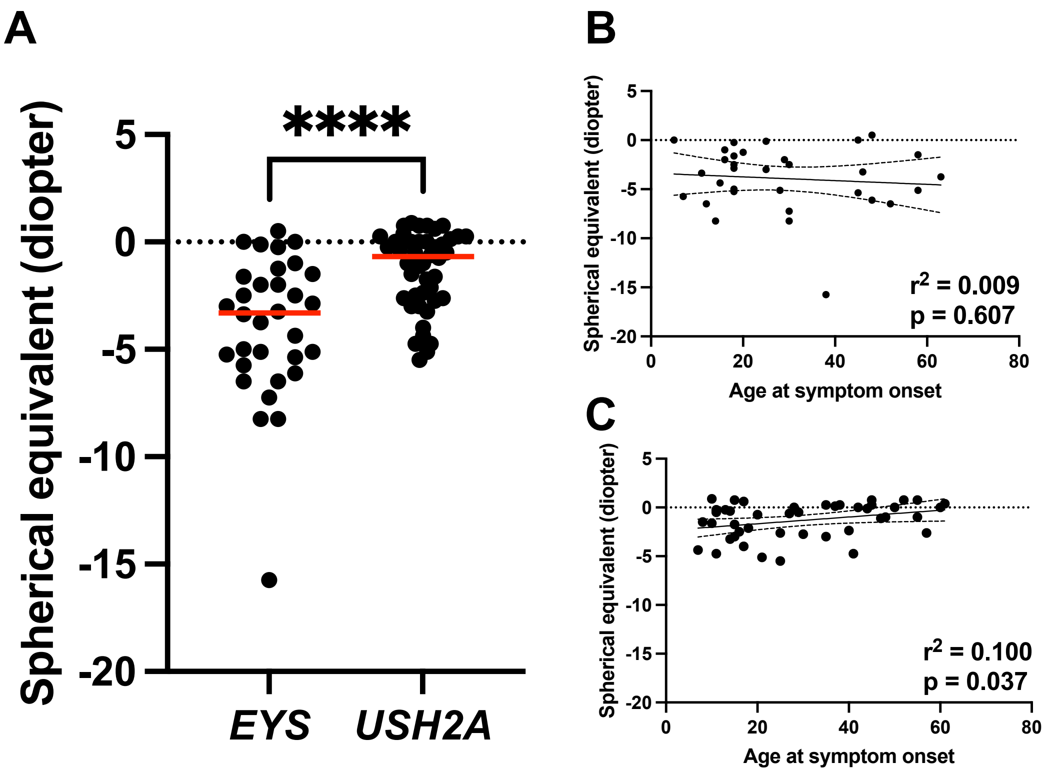

Supplement: Supplement 1 [file tvst-14-2-16_s001.pdf]
