# Supplementary material for: Phenotypic Distinctions Between EYS- and USH2A-Associated Retinitis Pigmentosa in an Asian Population
Source: Transl Vis Sci Technol. 2025 Feb 11;14(2):16. doi: 10.1167/tvst.14.2.16 (PMC11817848; doi:10.1167/tvst.14.2.16)

Supplementary Figure 2. Individuals with *EYS*-associated RP are more myopic than those with *USH2A*-associated RP, independent of gender. Boxes enclose the IQR and median spherical equivalent, with whiskers showing range. Subgroups were compared with a Kruskal Wallace test; *, p<0.05; ns, not significant.

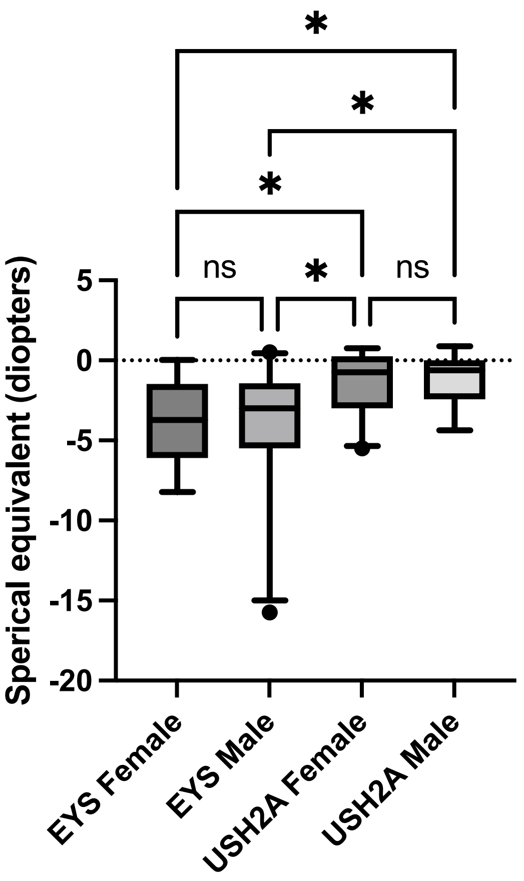

Supplement: Supplement 2 [file tvst-14-2-16_s002.pdf]
