# Supplementary material for: Phenotypic Distinctions Between EYS- and USH2A-Associated Retinitis Pigmentosa in an Asian Population
Source: Transl Vis Sci Technol. 2025 Feb 11;14(2):16. doi: 10.1167/tvst.14.2.16 (PMC11817848; doi:10.1167/tvst.14.2.16)
Supplement: Supplement 3 [file tvst-14-2-16_s003.pdf]

Supplementary Table 1. Genetic variants in *EYS* for the 35 individuals (n = 32 families) with nonsyndromic retinitis pigmentosa enrolled for the current study.

PATIENT	FAMILY	VARIANT 1	VARIANT 2
**IRD0042**		EYS c.7270_7271insCT p.F2424fs	EYS c.7492G>C p.A2498P
**IRD0064**		EYS c.6416G>A p.C2139Y	EYS c.8107G>T p.E2703X
**IRD0075**		EYS c.6416G>A p.C2139Y / c.8012T>A+ p.L2671X	EYS c.6416G>A p.C2139Y
**IRD0081**		EYS c.6416G>A p.C2139Y	EYS c.6416G>A p.C2139Y
**IRD0082**		EYS c.6416G>A p.C2139Y	EYS c.7228+1G>A
**IRD0091**		EYS c.8107G>T p.E2703X	EYS c.8107G>T p.E2703X
**IRD0137**	F013	EYS c.6416G>A p.C2139Y	EYS c.6416G>A p.C2139Y
**IRD0181**	F013	EYS c.6416G>A p.C2139Y	EYS c.6416G>A p.C2139Y
**IRD0143**		EYS c.6416G>A p.C2139Y	EYS c.8012T>A p.L2671X
**IRD0144**	F052	EYS c.6416G>A p.C2139Y	EYS c.5086G>T p.E1696X
**IRD0292**	F052	EYS c.6416G>A p.C2139Y	EYS c.5086G>T p.E1696X
**IRD0163**		EYS c.8107G>T p.E2703X	EYS c.8107G>T p.E2703X
**IRD0172**		EYS c.2831_2832del p.V944Gfs*9	EYS c.8860T>C p.F2954L
**IRD0177**		EYS c.7228+1G>A	EYS c.5644+5G>A
**IRD0186**		EYS c.8072-1G>A	EYS c.4408del p.E1470Kfs*8
**IRD0190**		EYS c.8107G>T p.E2703X	EYS c.4043C>T p.S1348F
**IRD0307**		EYS c.8243dup p.L2748Ffs*8	EYS c.103C>T p.Q35X
**IRD0324**	F027	EYS c.6416G>A p.C2139Y	EYS c.8309T>C p.L2770P
**IRD0325**	F027	EYS c.6416G>A p.C2139Y	EYS c.8309T>C p.L2770P
**IRD0355**		EYS c.6416G>A p.C2139Y	EYS c.7955del p.C2652Lfs*30
**IRD0413**		EYS c.6416G>A p.C2139Y	EYS c.8107G>T p.E2703X
**IRD0475**		EYS c.6416G>A p.C2139Y	EYS c.904C>T p.L302F
**IRD0484**		EYS c.7465T>G p.Y2489D	EYS c.6416G>A p.C2139Y
**IRD0539**		EYS c.8107G>T p.E2703X	EYS c.7492G>C p.A2498P
**IRD0555**		EYS c.6416G>A p.C2139Y / c.8012T>A+ p.L2671X	EYS c.904C>T p.L302F
**IRD0566**		EYS c.6416G>A p.C2139Y	EYS c.6416G>A p.C2139Y
**IRD0585**		EYS c.8860T>C p.F2954L	EYS c.6416G>A p.C2139Y
**IRD0589**		EYS c.6416G>A p.C2139Y	EYS c.5644+5G>A
**IRD0596**		EYS c.6416G>A p.C2139Y	EYS c.7941_7942del p.E2647Dfs*6
**IRD0861**		EYS c.2486delT p.I829TfsTer39	EYS c.2796T>A p.C932X
**IRD0916**		EYS c.3572G>A p.W1191X	EYS c.4807C>T p.Q1603X
**RP0073**		EYS c.6416G>A p.C2139Y	EYS c.8107G>T p.E2703X
**RP0131**		EYS c.6416G>A p.C2139Y	EYS c.8012T>A p.L2671X
**RP0286**		EYS c.6416G>A p.C2139Y / c.8012T>A+ p.L2671X	EYS c.5531del p.P1844Lfs*35
**RP0520**		EYS c.6416G>A p.C2139Y / c.8012T>A+ p.L2671X	EYS c.8107G>T p.E2703X
