# Supplementary material for: Phenotypic Distinctions Between EYS- and USH2A-Associated Retinitis Pigmentosa in an Asian Population
Source: Transl Vis Sci Technol. 2025 Feb 11;14(2):16. doi: 10.1167/tvst.14.2.16 (PMC11817848; doi:10.1167/tvst.14.2.16)
Supplement: Supplement 4 [file tvst-14-2-16_s004.pdf]

Supplementary Table 2. Genetic variants in *USH2A* for the 48 individuals (n = 44 families) with retinitis pigmentosa enrolled for the current study.

IRD#	FAMILY	ALLELE 1	ALLELE 2
**IRD0012**		USH2A c.15178T>C p.S5060P	USH2A c.2802T>G p.C934W
**IRD0013**		USH2A c.4576G>A p.G1526R	USH2A c.9790G>A p.G3264S
**IRD0015**		USH2A c.14133G>T p.Q4711H	USH2A c.14133G>T p.Q4711H
**IRD0025**		USH2A c.13339A>G p.M4447V / c.2653C>T p.H885Y	USH2A c.9469C>T p.Q3157X
**IRD0029**		USH2A c.12067-10_12067dup p.G4023Vfs*15	USH2A c.8559-2A>G
**IRD0030**		USH2A c.9570+1G>A	USH2A c.6929C>T p.T2310M
**IRD0039**		USH2A c.5858-1G>A	USH2A c.11506C>A p.P3836T
**IRD0046**		USH2A c.9570+1G>A	USH2A c.9570+1G>A
**IRD0059**		USH2A c.6485+1G>A	USH2A c.15178T>C p.S5060P
**IRD0067**		USH2A c.8906C>G p.S2969X	USH2A c.1877G>A p.R626Q
**IRD0084**		USH2A c.14218G>A p.A4740T	USH2A c.10342G>A p.E3448K
**IRD0095**		USH2A c.8232G>C p.W2744C	USH2A c.6923C>A p.A2308E
**IRD0113**		USH2A c.13339A>G p.M4447V	USH2A c.15178T>C p.S5060P
**IRD0117**		USH2A c.2802T>G p.C934W	USH2A c.449T>G p.L150X
**IRD0118**		USH2A c.7517A>G p.Y2506C	USH2A c.4400C>G p.P1467R
**IRD0153**	F135	USH2A c.11712-2A>C	USH2A c.2802T>G p.C934W
**IRD0615**	F135	USH2A c.11712-2A>C	USH2A c.2802T>G p.C934W
**IRD0162**		USH2A c.15178T>C p.S5060P	USH2A c.2792G>A p.C931Y
**IRD0171**		USH2A c.15178T>C p.S5060P	USH2A c.2802T>G p.C934W
**IRD0191**	F074	USH2A c.3665C>T p.A1222V	USH2A c.6050G>A p.G2017D
**IRD0335**	F074	USH2A c.3665C>T p.A1222V	USH2A c.6050G>A p.G2017D
**IRD0200**		USH2A c.7076T>C p.L2359S	USH2A c.2802T>G p.C934W
**IRD0201**		USH2A c.8559-2A>G	USH2A c.1173T>A p.S391R
**IRD0222**		USH2A c.13339A>G p.M4447V	USH2A c.5051C>T p.P1684L
**IRD0246**		USH2A c.9469C>T p.Q3157X	USH2A c.449T>G p.L150X
**IRD0247**		USH2A c.13916A>C p.H4639P	USH2A c.2802T>G p.C934W / c.5953G>A p.E1985K
**IRD0306**		USH2A c.12066+1G>C	USH2A c.907C>T p.R303C
**IRD0330**		USH2A c.13339A>G p.M4447V	USH2A c.1181C>G p.P394R
**IRD0340**	F106	USH2A c.2802T>G p.C934W	USH2A c.2802T>G p.C934W
**RP0034**	F106	USH2A c.2802T>G p.C934W	USH2A c.2802T>G p.C934W
**IRD0348**	F030	USH2A c.8559-2A>G	USH2A c.2802T>G p.C934W
**IRD0390**	F030	USH2A c.8559-2A>G	USH2A c.2802T>G p.C934W
**IRD0369**		USH2A c.13339A>G p.M4447V	USH2A c.14201C>T p.P4734L
**IRD0400**		USH2A c.2802T>G p.C934W	USH2A c.12361C>T p.R4121C / c.13121T>A p.V4374E
**IRD0409**		USH2A c.2802T>G p.C934W	USH2A c.11712-2A>C
**IRD0451**		USH2A c.5252G>T p.G1751V	USH2A c.5012G>A p.G1671D
**IRD0463**		USH2A c.7831T>C p.S2611P	USH2A c.1824dup p.E609X
**IRD0477**		USH2A c.14798A>C p.Q4933P	USH2A c.11712-2A>C
**IRD0498**		USH2A c.1181C>G p.P394R	USH2A c.13339A>G p.M4447V
**IRD0516**		USH2A c.11156G>A p.R3719H	USH2A c.5581G>A p.G1861S

**IRD0541**	USH2A c.13339A>G p.M4447V	USH2A c.14453C>T p.P4818L
**IRD0571**	USH2A c.2802T>G p.C934W	USH2A c.9469C>T p.Q3157X
**IRD0575**	USH2A c.99_100insT p.R34fs	USH2A c.151A>T p.I51F
**IRD0584**	USH2A c.2802T>G p.C934W / c.7068T>G p.N2356K	USH2A c.2187C>A p.C729X / c.997T>C p.S333P
**IRD0609**	USH2A c.2802T>G p.C934W	USH2A c.8559-2A>G
**IRD0611**	USH2A c.9570+1G>A	USH2A c.8559-2A>G
**IRD0804**	USH2A c.4576G>A p.G1526R	USH2A c.7919T>A p.V2640E
**RP0086**	USH2A c.13649T>G p.V4550G	USH2A c.5788C>T p.R1930X
