# Supplementary material for: Phenotypic Distinctions Between EYS- and USH2A-Associated Retinitis Pigmentosa in an Asian Population
Source: Transl Vis Sci Technol. 2025 Feb 11;14(2):16. doi: 10.1167/tvst.14.2.16 (PMC11817848; doi:10.1167/tvst.14.2.16)
Supplement: Supplement 5 [file tvst-14-2-16_s005.pdf]

Supplementary Table 3. Genetic variants in the secondary study cohort from Nagoya, Japan. A total of 10 patients with *EYS*- and 9 patients with *USH2A*-associated retinitis pigmentosa were used to assess utility of clinical biomarkers.

PATIENT	FAMILY	VARIANT 1	VARIANT 2
**N-71**		*EYS* c.4957dupA p.Ser1653fs	*EYS* c.4957dupA p.Ser1653fs
**N-114**		*EYS* c.8805C>A p.Tyr2935*	*EYS* c.2528G>A p.Gly843Glu
**N-167**		*EYS* c.4957dupA p.Ser1653fs	*EYS* c.4957dupA p.Ser1653fs
**N-204**		*EYS* c.6563T>C p.Ile2188Thr	*EYS* c.6557G>A p.Gly2186Glu
**N-224**		*EYS* c.7665_7666delCA p.Tyr2555fs	*EYS* c.2528G>A p.Gly843Glu
**N-228**		*EYS* c.8805C>A p.Tyr2935*	*EYS* c.6557G>A p.Gly2186Glu
**N-240**		*EYS* c.4957dupA p.Ser1653fs	*EYS* c.2528G>A p.Gly843Glu
**N-251**		*EYS* c.4957dupA p.Ser1653fs	*EYS* c.2528G>A p.Gly843Glu
**N-274**		*EYS* c.4957dupA p.Ser1653fs	*EYS* c.2528G>A p.Gly843Glu
**N-291**		*EYS* c.2528G>A p.Gly843Glu	*EYS* c.2528G>A p.Gly843Glu
**N-75**		*USH2A* c.13112_13115delAAAT p.Gln4371fs	*USH2A* c.2802T>G p.Cys934Trp
**N-109**		*USH2A* c.14243C>T p.Ser4748Phe	*USH2A* c.2802T>G p.Cys934Trp
**N-168**		*USH2A* c.10999A>C p.Thr3667Pro	*USH2A* c.490G>T p.Val164Phe
**N-294**		*USH2A* c.2802T>G p.Cys934Trp	*USH2A* c.2802T>G p.Cys934Trp
**N-1023**		*USH2A* c.8254G>A p.Gly2752Arg	*USH2A* c.2802T>G p.Cys934Trp
**N-1152**		*USH2A* c.12416delG p.Gly4139fs	*USH2A* c.490G>T p.Val164Phe
**N-524**	1	*USH2A* c.8254G>A p.Gly2752Arg	*USH2A* c.3596_3598delAAG p.Glu1199del
**N-712**	1	*USH2A* c.8254G>A p.Gly2752Arg	*USH2A* c.3596_3598delAAG p.Glu1199del
**N-771**		*USH2A* c.10999A>C p.Thr3667Pro	*USH2A* c.5865_5866delAA p.Ser1956fs
