# Supplementary material for: Phenotypic Distinctions Between EYS- and USH2A-Associated Retinitis Pigmentosa in an Asian Population
Source: Transl Vis Sci Technol. 2025 Feb 11;14(2):16. doi: 10.1167/tvst.14.2.16 (PMC11817848; doi:10.1167/tvst.14.2.16)
Supplement: Supplement 6 [file tvst-14-2-16_s006.pdf]

Supplementary Table 4. Logistic regression analysis of *EYS*- and nonsyndromic *USH2A*-associated RP, showing the influence of genotype, EZ band width, and patient age, on the presence of a parafoveal ring on fundus autofluorescence. *USH2A* genotype was independently associated with a parafoveal ring (*), independent of patient age and ellipsoid band width.

Variable	Coefficient (β)	Standard Error (SE)	z-value	p-value	95% Confidence Interval
Intercept	0.628	0.979	0.641	0.522	–1.292, 2.547
USH2A genotype	2.048	0.595	3.443	0.001*	0.882, 3.214
EZ Width	–0.0002	0.000	–1.853	0.064	–0.000, 1.26e-05
Age	–0.018	0.020	-0.859	0.391	–0.058, 0.023
