# Supplementary material for: Phenotypic Distinctions Between EYS- and USH2A-Associated Retinitis Pigmentosa in an Asian Population
Source: Transl Vis Sci Technol. 2025 Feb 11;14(2):16. doi: 10.1167/tvst.14.2.16 (PMC11817848; doi:10.1167/tvst.14.2.16)
Supplement: Supplement 7 [file tvst-14-2-16_s007.pdf]

Supplementary Table 5. Logistic regression analysis of *EYS*- and nonsyndromic *USH2A*-associated RP, showing the influence of genotype, EZ band width, and patient age, on the presence of peripapillary nasal sparing on fundus autofluorescence. *EYS* genotype and ellipsoid band length were independently associated with nasal sparing (*).

**Variable**	**Coefficient (β)**	**Standard Error (SE)**	**z-value**	**p-value**	**95% Confidence Interval**
Intercept	–1.677	1.206	–1.391	0.164	–4.04, 0.686
EYS genotype	1.796	0.641	2.802	0.005*	0.54, 3.052
EZ Width	0.0005	0.0001	3.352	0.001*	0.0002, 0.0008
Age	–0.023	0.021	–1.085	0.278	–0.065, 0.019
