# Supplementary material for: Phenotypic Distinctions Between EYS- and USH2A-Associated Retinitis Pigmentosa in an Asian Population
Source: Transl Vis Sci Technol. 2025 Feb 11;14(2):16. doi: 10.1167/tvst.14.2.16 (PMC11817848; doi:10.1167/tvst.14.2.16)
Supplement: Supplement 8 [file tvst-14-2-16_s008.pdf]

Supplementary Table 6. Multiple logistic regression estimates for clinical features that distinguish between *EYS*- and *USH2A*-associated retinitis pigmentosa. Area under the ROC curve was 0.832 (SE 0.048; 95% C.I. 0.738, 0.925), with a p value of <0.0001. The logistic regression model had a negative predictive power of 77.8% and positive predictive power of 80.0%.

Parameter estimates	***Variable***	***Estimate***	***Standard error***	***95% CI (profile likelihood)***
β0	Intercept	–0.750	0.682	–2.171 to 0.554
β1	Age at onset <28 yrs	0.149	0.612	–1.058 to 1.377
β2	Presenting BCVA <0.2	1.215	0.721	–0.128 to 2.743
β3	Spherical equivalent <–0.2 D	1.527	0.606	0.367 to 2.775
β4	Nasal peripapillary sparing	1.298	0.638	0.082 to 2.619
β5	Parafoveal ring	–1.670	0.611	–2.931 to -0.506
